# Combining STAT3-Targeting Agents with Immune Checkpoint Inhibitors in NSCLC

**DOI:** 10.3390/cancers15020386

**Published:** 2023-01-06

**Authors:** Kostas A. Papavassiliou, Georgios Marinos, Athanasios G. Papavassiliou

**Affiliations:** 1 First University Department of Respiratory Medicine, Medical School, “Sotiria” Hospital, National and Kapodistrian University of Athens, 11527 Athens, Greece; 2 Department of Hygiene, Epidemiology and Medical Statistics, Medical School, National and Kapodistrian University of Athens, 11527 Athens, Greece; 3 Department of Biological Chemistry, Medical School, National and Kapodistrian University of Athens, 11527 Athens, Greece

**Keywords:** NSCLC, STAT3, drug resistance, immune checkpoint inhibitors

## Abstract

**Simple Summary:**

Signal transducer and activator of transcription 3 (STAT3) plays a critical role in the development and progression of non-small cell lung cancer (NSCLC) tumors. Several reports suggest that STAT3 is involved in immunosuppression and the promotion of resistance to immune checkpoint inhibitors. In this review, we discuss the potential therapeutic strategy of combining STAT3 inhibition with immune checkpoint inhibitors to overcome drug resistance and enhance their efficacy in NSCLC.

**Abstract:**

Despite recent therapeutic advances, non-small cell lung cancer (NSCLC) remains the leading cause of cancer-related death. Signal transducer and activator of transcription 3 (STAT3) is a transcription factor (TF) with multiple tumor-promoting effects in NSCLC, including proliferation, anti-apoptosis, angiogenesis, invasion, metastasis, immunosuppression, and drug resistance. Recent studies suggest that STAT3 activation contributes to resistance to immune checkpoint inhibitors. Thus, STAT3 represents an attractive target whose pharmacological modulation in NSCLC may assist in enhancing the efficacy of or overcoming resistance to immune checkpoint inhibitors. In this review, we discuss the biological mechanisms through which STAT3 inhibition synergizes with or overcomes resistance to immune checkpoint inhibitors and highlight the therapeutic strategy of using drugs that target STAT3 as potential combination partners for immune checkpoint inhibitors in the management of NSCLC patients.

## 1. Introduction

Lung cancer is one of the most aggressive cancers and is responsible for the majority of cancer deaths worldwide [1]. It is mainly divided into non-small cell lung cancer (NSCLC) and small cell lung cancer (SCLC), with NSCLC accounting for 85% of all lung cancer cases. Although patients with a localized disease have a favorable prognosis, most patients present at advanced stages when first diagnosed and have a poor prognosis [2].

Recent advances in molecular profiling technologies have deepened our understanding of NSCLC’s biology. This knowledge translated into the development and introduction of novel targeted therapies and immunotherapies that have dramatically changed the management of patients with advanced NSCLC, leading to improved survival outcomes. Nevertheless, the vast majority of advanced NSCLCs do not respond to or become resistant to current treatments and eventually worsen. Thus, there is an urgent need to identify new therapeutic targets that can be modulated in order to overcome resistance to or enhance the efficacy of standard treatments. Potential strategies may involve the co-targeting of other immune checkpoints or the targeting of upregulated oncogenic signaling pathways that induce an immunosuppressive tumor microenvironment.

Transcription factors (TFs) are vital proteins that orchestrate gene expression and are located at the bottleneck of signaling pathways. All cancers exploit TFs by deregulating them to promote their malignant characteristics, and NSCLC is no exception. STAT3 is an important TF that is aberrantly activated in NSCLC and is associated with a poor clinical prognosis [3]. STAT3 signaling is involved in multiple cancer cell hallmarks, including proliferation, anti-apoptosis, angiogenesis, invasion and metastasis, immunosuppression, and drug resistance [4].

Herein, we chose to focus on STAT3 because it is a key oncogenic TF involved in antitumor immune response mechanisms, and research on STAT3 and NSCLC has revealed that this TF participates in mechanisms of drug resistance to immune checkpoint inhibitors. Dissecting these mechanisms and developing agents that target STAT3 may allow immune checkpoint inhibitors to overcome drug resistance and achieve their full potential in the clinic.

## 2. STAT3 Transcription Factor and STAT3 Signaling

The TF STAT3 is a member of the STAT family of proteins composed of seven proteins, namely, STAT1, STAT2, STAT3, STAT4, STAT5A, STAT5B, and STAT6 [5]. Structurally, STAT3 has a conserved N-terminal domain, a coiled-coil domain, a DNA-binding domain, a linker domain, an SH2 domain used for dimerization and receptor binding, and a C-terminal transactivation domain used for interaction with cofactors [5]. Several growth factors, cytokines, and hormones are able to activate STAT3 when bound to cytokine receptors, such as the interleukin 6 receptor (IL-6R) and interleukin 10 receptor (IL-10R), and growth factor receptors, such as epidermal growth factor receptor (EGFR), insulin-like growth factor receptor (IGFR), and fibroblast growth factor receptor (FGFR). Ligand binding triggers receptor dimerization, recruitment of glycoprotein 130 (gp130), and Janus kinases (JAKs), which results in the phosphorylation and activation of JAKs [6]. In turn, the activated JAKs phosphorylate the tyrosine residues located at the cytoplasmic side of the receptors, which allows for the STAT3-related SH2 domain to interact with them and lead to the JAK-mediated phosphorylation of STAT3 [7]. STAT3 phosphorylation and activation can also occur via receptor-independent tyrosine kinases, such as Abl and c-SRC [8]. When phosphorylated, STAT3 forms homodimers which translocate from the cytoplasm to the nucleus. Once inside the nucleus, activated STAT3 dimers interact with coactivators, such as CREB-binding protein (CBP) and apurinic/apyrimidinic endonuclease-1 (APE), and bind to specific DNA sequences upregulating the transcription of target genes [9,10]. Under normal conditions, in order to tightly regulate the function of STAT3, normal cells use endogenous inhibitors, including the protein inhibitor of activated STAT3 (PIAS), multiple protein tyrosine phosphatases (PTPs), and the suppressor of cytokine signaling (SOCS) [11] (see also figure and corresponding caption at https://www.ncbi.nlm.nih.gov/pmc/articles/PMC3355894/figure/F1/ [12], accessed on 30 December 2022).

## 3. The Role of STAT3 in NSCLC

The tumor samples of most NSCLC patients display persistently activated STAT3, and this upregulated expression correlates with an advanced clinical stage, metastasis to lymph nodes, and drug resistance [13,14,15,16,17,18]. Constitutive activation of STAT3 in NSCLC occurs via several mechanisms: (1) mutated receptor tyrosine kinases (e.g., EGFR) and receptor-independent tyrosine kinases (e.g., SRC), (2) increased cytokine and growth factor levels within the tumor that can activate STAT3, and (3) deregulation of the endogenous STAT3 inhibitors, including PTPs, PIAS, and SOCS [18,19,20,21,22]. Studies have identified a key oncogenic role for STAT3 in NSCLC: participating in the promotion of many cancer hallmarks, such as immune suppression, cell survival, angiogenesis, drug resistance, and cancer cell stemness [4].

### STAT3-Based Immunosuppression

The tumor microenvironment is an important aspect of cancer and acts in concert with cancer cells to promote their malignant growth. In NSCLC, STAT3 activation within cancer or immune cells (Table 1) favors an immunosuppressive tumor microenvironment and supports an inflammatory state that promotes tumor growth. Here, we will focus on the oncogenic effects of STAT3 when activated within immune cells.

When STAT3 is overexpressed in alveolar type II cells, severe pulmonary inflammation takes place, which results in the spontaneous formation of lung adenocarcinoma in mice [23]. In a lung adenocarcinoma model induced by the carcinogen urethane, genetic ablation of STAT3 from the lung epithelium led to an increased production of pro-inflammatory cytokines and enhanced function of natural killer (NK) cells that substantially inhibited urethane-induced lung carcinogenesis [24]. The silencing of STAT3 in a human cellular model of NSCLC upregulated the production of pro-inflammatory chemokines and made NSCLC cells more susceptible to the cytotoxic function of NK cells [24].

In the lung, when STAT3 is aberrantly activated within myeloid cells, it promotes the development of cancer by recruiting the tumor microenvironment immune cells that have immunosuppressive functions. In contrast, STAT3 deletion in myeloid cells results in the generation of NK and cytotoxic T-cells that respond effectively and hinder tumor growth [25]. In macrophages, activated STAT3 increases M2 polarization, programmed cell death ligand 1 (PD-L1) expression, immune suppression, and angiogenesis [26,27,28,29]. In myeloid-derived suppressor cells (MDSCs), STAT3 activation promotes their development and proliferation, upregulates PD-L1 expression, and results in immune suppression [30,31]. In dendritic cells, activated STAT3 inhibits their maturation, activation, and the presentation of antigens [32,33,34]. In neutrophils, the activation of STAT3 leads to defective chemotaxis, decreased cytokine production, and cytolytic activity [35,36,37]. In regulatory T-cells, STAT3 activation promotes their expansion and differentiation and upregulates the expression of the immune checkpoint molecule cytotoxic T-lymphocyte associated protein 4 (CTLA4) [38,39,40]. Activation of STAT3 in B-cells promotes their proliferation and survival, as well as angiogenesis and immunosuppression [41,42,43,44,45]. In NK and T-cells, activated STAT3 inhibits their activation, proliferation, chemotaxis, and cytotoxicity [24,46,47,48,49].

## 4. Immune Checkpoint Molecules and Their Targeting in NSCLC

The human immune system can locate, recognize, and eliminate cancer cells, a process that is regulated by inhibitory and activating proteins. Cancer cells often use immune checkpoint molecules, such as programmed cell death protein 1 (PD-1)/PD-L1 (CD274), lymphocyte activation gene 3 (LAG3, CD223), CTLA4, hepatitis A virus cellular receptor 2 (HAVCR2, TIM3), and T-cell immunoreceptor with Ig and ITIM domains (TIGIT), to suppress the immune system and continue their malignant growth [50,51]. This interaction between cancer and immune cells can be reversed and blocked via the use of specific antibodies that are called immune checkpoint inhibitors. PD-L1 protein levels have been found to be upregulated in NSCLC patients, and nowadays, checkpoint inhibitors that block the interaction between PD-1 and PD-L1 are routinely used in their clinical management [52,53,54,55]. Despite the increased expression of immune checkpoint molecules, such as PD-L1, in NSCLCs where the growth relies on mutated oncogenes (oncogene addicted), therapy with only immune checkpoint inhibitors has not generated promising results due to intrinsic or acquired resistance [56,57]. Intrinsic resistance can be due to genetic and epigenetic alterations in cancer cells related to immune recognition, signaling pathways, gene expression, and DNA damage responses, while extrinsic mechanisms involve the interaction of cancer cells with stromal cells, immune cells, or even the microbiome. In order to develop a therapeutic strategy based on immune checkpoint blockade that will be more effective and durable for NSCLC patients, we must understand the regulatory mechanisms of immune checkpoint molecules.

## 5. STAT3 and PD-L1 in NSCLC

At the transcriptional level, PD-L1 is controlled by a variety of TFs, including, among others, STAT3, Myc, nuclear factor kappa-B (NF-κB), and hypoxia-inducible factor 1 subunit alpha (HIF-1α) [58]. By directly binding to the promoter of PD-L1, the S STAT3 regulates PD-L1 expression in a plethora of cancers, such as T-cell lymphoma [59], breast cancer [60], nasopharyngeal carcinoma [61], osteosarcoma [62], and gastric cancer [63]. Several studies have identified an association between STAT3 and PD-L1 in NSCLC (Table 2).

In the biological setting of NSCLC, mutated EGFR signaling phosphorylates and activates STAT3, which in turn induces the upregulation of PD-L1 expression [64]. The same mechanism occurs in NSCLCs that harbor ALK chromosomal rearrangements [65,66,67]. In NSCLC with mutated KRAS, STAT3 was found to participate in the regulation of PD-L1 expression, and the silencing of STAT3 led to reduced PD-L1 expression [68]. Moreover, miR-3127-5p-mediated STAT3 activation upregulated the expression of PD-L1 and resulted in the promotion of immune escape and resistance to chemotherapy [69]. In EGFR-mutant NSCLC that is resistant to the tyrosine kinase inhibitor gefitinib, activation of STAT3 caused the upregulation of PD-L1 expression while its inhibition led to the downregulation of PD-L1. Interestingly, the use of niclosamide, an antihelmintic drug, when combined with immune checkpoint inhibitors in vitro and in vivo led to the enhancement of the PD-L1 blockade, delaying tumor growth. This effect was shown to be brought about via blocking the binding of STAT3 to the promoter of PD-L1 [70,71]. Another recent study found that NSCLC patients with mutated serine/threonine kinase 11 (STK11) do not respond favorably to the combined treatment with immune checkpoint inhibitors against PD-L1 (durvalumab) and CTLA4 (tremelimumab). This resistance was attributed to the increased expression of genes and cytokines that activate STAT3 signaling in lung tumors. Of note, in vivo data showed that STAT3 knockdown reversed this resistance, implying that STAT3-targeting, together with immune checkpoint inhibitors, is a viable pharmacological strategy that can overcome resistance to immunotherapy in NSCLC [72]. In addition to STAT3-mediated PD-L1 upregulation in cancer cells, activated STAT3 also upregulates the expression of immune checkpoint molecules on the cell surface of immune cells, including regulatory T-cells [39], B-cells [73], CD4 [74], CD8 [75], and MDSCs [29]. It is evident from the above findings that STAT3 plays an important role in immunosuppression by regulating the expression of the immune checkpoint protein PD-L1 in both cancer and immune cells and may be a promising therapeutic target that can be modulated in order to overcome resistance to and enhance the efficacy of immune checkpoint inhibitors in NSCLC. As a proof of concept, there are currently three clinical trials underway that are evaluating the combination of STAT3 inhibition together with immune checkpoint inhibitors against PD-L1 (Table 3).

In contrast to what was presented above, a downregulation in the expression of PD-L1 after STAT3 inhibition might not enhance but rather decrease the efficacy of immune checkpoint inhibitors. A recent study in melanoma cases identified *JAK1* and *JAK2* mutations that were associated with acquired resistance to PD-L1 blockade with pembrolizumab [76]. In the setting of NSCLC, another study found *JAK2*-inactivating mutations that were correlated with a downregulation in the expression of PD-L1 [77]. *JAK2* mutations have been found to be more common in lung adenocarcinomas among African American populations [78]; thus, it is essential to evaluate the effect of these mutations on the response to immune checkpoint inhibitors. Based on these studies, it can be reasoned that the effect of pharmacologically blocking STAT3 would resemble that of the *JAK*-inactivating mutations.

## 6. Concluding Remarks and Future Perspectives

NSCLC still remains a fatal disease, and most patients present with an advanced stage that has a poor prognosis. Current therapeutic options, including immune checkpoint inhibitors, have provided promising results; however, most patients relapse or do not respond to treatment. There is an urgent need to discover novel targets that can assist in overcoming drug resistance or enhance the efficacy of standard treatments. The tumor microenvironment acts in concert with NSCLC cells to promote their malignancy, and STAT3 signaling functions as a mediator of external signals emanating from nearby immune cells or extracellular mechanical signals [79]. Upstream signals in cancer cells lead to the activation of STAT3, which in turn induces the upregulation of the expression of the immune checkpoint molecule PD-L1 in several cancer types (e.g., [80]), including NSCLC. STAT3 signaling also influences the immune microenvironment by suppressing the function of immune cells. Given the important role of the TF STAT3 in promoting immunosuppression and resistance to immune checkpoint inhibitors, STAT3 represents a potential target that can be modulated via different targeting strategies [81] and combined with immunotherapies in cancer. By following this therapeutic strategy, the same immune checkpoint pathway is being targeted at two different but vital nodes and thus leaves very little room for cancer cells to develop resistance to this treatment combination. Similarly to NSCLC, this drug combination appears to be working in settings of other cancer types as well [82,83] and has led to the initiation of clinical trials [84]. In addition to immune checkpoint inhibitors, blockade of STAT3 signaling may also enhance the efficacy of other types of immunotherapies. Should STAT3-targeting agents be combined with immune checkpoint inhibitors for the management of NSCLC in the future, it is essential to know which particular patients are more likely to respond to this therapeutic combination via the use of suitable predictive biomarkers. Preclinical data suggest that activated STAT3, as well as STAT3 mRNA expression, may be utilized as predictive biomarkers for immune checkpoint inhibitors in oncogene-addicted NSCLC patients [85]. Along the same vein as the subject of this review, gene ontology and clustering analysis using the Database for Annotation, Visualization, and Integrated Discovery (DAVID) and BioLattice revealed that triple-negative breast cancer (TNBC) patients with highly suspicious microcalcifications on mammography are strongly associated with decreased immune system activity [86,87]. Notably, pyroptosis-mediated pattern clusters may be used to determine the risk score of a breast tumor for individual patients, thus helping predict the prognosis and effectiveness of combinatorial treatments employing immune checkpoint inhibitors [88]. On the other hand, studies have suggested that histone deacetylase inhibitors (HDACi) could upregulate PD-L1 expression in tumor cells and change the state of the anti-tumor immune responses in vivo. To this end, it has been recently shown that FK228, a HDACi exhibiting anti-tumor effects against several types of solid tumors [89], in combination with anti-PD-1 immunotherapy, provides a more suitable treatment for colon cancer [90]. It remains to be seen whether co-targeting STAT3 and immune checkpoint molecules in NSCLC will prove to be a successful therapeutic strategy.

## Figures and Tables

**Table 1:** Cancer-related effects of activated STAT3 in immune cells.

Immune Cells	Effects
Macrophages	Increases M2 polarization, PD-L1 expression, immune suppression, angiogenesis
MDSCs	Promotes cell development, proliferation, upregulates PD-L1 expression, promotes immunosuppression
Dendritic cells	Inhibits cell maturation, activation, antigen presentation
Neutrophils	Defective chemotaxis and cytolytic activity, decreased cytokine production
Regulatory T-cells	Cell expansion and differentiation, upregulates CTLA4 expression
B-cells	Promotes cell proliferation and survival, angiogenesis, immunosuppression
Natural killer and T-cells	Inhibits cell proliferation, activation, chemotaxis, cytotoxicity

MDSCs, myeloid-derived suppressor cells; PD-L1, programmed cell death ligand 1; CTLA4, cytotoxic T-lymphocyte associated protein 4.

**Table 2:** STAT3-related mechanisms of PD-L1 regulation in NSCLC.

NSCLC Setting	Mechanism of PD-L1 Upregulation
EGFR-mutant	Activation of IL-6/JAK/STAT3 axis
ALK chromosomal rearrangements	Activation of IL-6/JAK/STAT3 axis
KRAS-mutant	Activation of RAS/MAPK/STAT3 axis
EGFR-mutant (gefitinib-resistant)	Activation of AKT/STAT3 axis
STK11-mutant	Upregulation of genes and cytokines that activate STAT3

EGFR, epidermal growth factor receptor; ALK, anaplastic lymphoma kinase; STK11, serine/threonine kinase 11; IL-6, interleukin 6; JAK, Janus kinase; MAPK, mitogen-activated protein kinase.

**Table 3:** Clinical trials combining STAT3-targeting agents with immune checkpoint inhibitors in NSCLC.

Mechanism of Action	Drug Combination	Status	Phase	NCT #
Degradation of STAT3 mRNA	AZD9150 and Durvalumab	Active	II	NCT02983578
Degradation of STAT3 mRNA	AZD9150 and Durvalumab	Recruiting	II	NCT03334617
Degradation of STAT3 mRNA	AZD9150 and Durvalumab	Active	I/II	NCT03421353

NCT, National Clinical Trial.

## References

[1] Sung H., Ferlay J., Siegel R.L., Laversanne M., Soerjomataram I., Jemal A., Bray F. (2021). Global Cancer Statistics 2020: GLOBOCAN Estimates of Incidence and Mortality Worldwide for 36 Cancers in 185 Countries. CA Cancer J. Clin..

[2] Walters S., Maringe C., Coleman M.P., Peake M.D., Butler J., Young N., Bergström S., Hanna L., Jakobsen E., Kölbeck K. (2013). Lung cancer survival and stage at diagnosis in Australia, Canada, Denmark, Norway, Sweden and the UK: A population-based study, 2004–2007. Thorax.

[3] Xu Y.H., Lu S. (2014). A meta-analysis of STAT3 and phospho-STAT3 expression and survival of patients with non-small-cell lung cancer. Eur. J. Surg. Oncol. J. Eur. Soc. Surg. Oncol. Br. Assoc. Surg. Oncol..

[4] Parakh S., Ernst M., Poh A.R. (2021). Multicellular Effects of STAT3 in Non-small Cell Lung Cancer: Mechanistic Insights and Therapeutic Opportunities. Cancers.

[5] Levy D.E., Darnell J.E. (2002). Stats: Transcriptional control and biological impact. Nat. Rev. Mol. Cell. Biol..

[6] Garbers C., Aparicio-Siegmund S., Rose-John S. (2015). The IL-6/gp130/STAT3 signaling axis: Recent advances towards specific inhibition. Curr. Opin. Immunol..

[7] Johnson D.E., O’Keefe R.A., Grandis J.R. (2018). Targeting the IL-6/JAK/STAT3 signalling axis in cancer. Nat. Rev. Clin. Oncol..

[8] Karras J.G., Wang Z., Huo L., Howard R.G., Frank D.A., Rothstein T.L. (1997). Signal transducer and activator of transcription-3 (STAT3) is constitutively activated in normal, self-renewing B-1 cells but only inducibly expressed in conventional B lymphocytes. J. Exp. Med..

[9] Yu H., Lee H., Herrmann A., Buettner R., Jove R. (2014). Revisiting STAT3 signalling in cancer: New and unexpected biological functions. Nat. Rev. Cancer.

[10] Guanizo A.C., Fernando C.D., Garama D.J., Gough D.J. (2018). STAT3: A multifaceted oncoprotein. Growth Factors.

[11] Morris R., Kershaw N.J., Babon J.J. (2018). The molecular details of cytokine signaling via the JAK/STAT pathway. Protein Sci..

[12] Nair R.R., Tolentino J.H., Hazlehurst L.A. (2012). Role of STAT3 in transformation and drug resistance in CML. Front. Oncol..

[13] Haura E.B., Zheng Z., Song L., Cantor A., Bepler G. (2005). Activated epidermal growth factor receptor-Stat-3 signaling promotes tumor survival in vivo in non-small cell lung cancer. Clin. Cancer Res..

[14] Sánchez-Ceja S.G., Reyes-Maldonado E., Vázquez-Manríquez M.E., López-Luna J.J., Belmont A., Gutiérrez-Castellanos S. (2006). Differential expression of STAT5 and Bcl-xL, and high expression of Neu and STAT3 in non-small-cell lung carcinoma. Lung Cancer.

[15] Yin Z., Zhang Y., Li Y., Lv T., Liu J., Wang X. (2012). Prognostic significance of STAT3 expression and its correlation with chemoresistance of non-small cell lung cancer cells. Acta Histochem..

[16] Yin Z.J., Jin F.G., Liu T.G., Fu E.Q., Xie Y.H., Sun R.L. (2011). Overexpression of STAT3 potentiates growth, survival, and radioresistance of non-small-cell lung cancer (NSCLC) cells. J. Surg. Res..

[17] Lee H.J., Zhuang G., Cao Y., Du P., Kim H.J., Settleman J. (2014). Drug resistance via feedback activation of Stat3 in oncogene-addicted cancer cells. Cancer Cell.

[18] Jiang R., Jin Z., Liu Z., Sun L., Wang L., Li K. (2011). Correlation of Activated STAT3 Expression with Clinicopathologic Features in Lung Adenocarcinoma and Squamous Cell Carcinoma. Mol. Diagn. Ther..

[19] Kim S.M., Kwon O.J., Hong Y.K., Kim J.H., Solca F., Ha S.J., Soo R.A., Christensen J.G., Lee J.H., Cho B.C. (2012). Activation of IL-6R/JAK1/STAT3 signaling induces de novo resistance to irreversible EGFR inhibitors in non-small cell lung cancer with T790M resistance mutation. Mol. Cancer.

[20] Wang X., Chen Z. (2015). MicroRNA-19a functions as an oncogenic microRNA in non-small cell lung cancer by targeting the suppressor of cytokine signaling 1 and mediating STAT3 activation. Int. J. Mol. Med..

[21] Im J.-Y., Kim B.-K., Lee K.-W., Chun S.-Y., Kang M.-J., Won M. (2020). DDIAS promotes STAT3 activation by preventing STAT3 recruitment to PTPRM in lung cancer cells. Oncogenesis.

[22] Kluge A., Dabir S., Vlassenbroeck I., Eisenberg R., Dowlati A. (2011). Protein inhibitor of activated STAT3 expression in lung cancer. Mol. Oncol..

[23] Li Y., Du H., Qin Y., Roberts J., Cummings O.W., Yan C. (2007). Activation of the signal transducers and activators of the transcription 3 pathway in alveolar epithelial cells induces inflammation and adenocarcinomas in mouse lung. Cancer Res..

[24] Ihara S., Kida H., Arase H., Tripathi L.P., Chen Y.A., Kimura T., Yoshida M., Kashiwa Y., Hirata H., Fukamizu R. (2012). Inhibitory roles of signal transducer and activator of transcription 3 in antitumor immunity during carcinogen-induced lung tumorigenesis. Cancer Res..

[25] Zhou J., Qu Z., Sun F., Han L., Li L., Yan S., Stabile L.P., Chen L.F., Siegfried J.M., Xiao G. (2017). Myeloid STAT3 Promotes Lung Tumorigenesis by Transforming Tumor Immunosurveillance into Tumor-Promoting Inflammation. Cancer Immunol. Res..

[26] Giurisato E., Xu Q., Lonardi S., Telfer B., Russo I., Pearson A., Finegan K.G., Wang W., Wang J., Gray N.S. (2018). Myeloid ERK5 deficiency suppresses tumor growth by blocking protumor macrophage polarization via STAT3 inhibition. Proc. Natl. Acad. Sci. USA.

[27] Sun L., Chen B., Jiang R., Li J., Wang B. (2017). Resveratrol inhibits lung cancer growth by suppressing M2-like polarization of tumor associated macrophages. Cell. Immunol..

[28] Yuan F., Fu X., Shi H., Chen G., Dong P., Zhang W. (2014). Induction of murine macrophage M2 polarization by cigarette smoke extract via the JAK2/STAT3 pathway. PLoS ONE.

[29] Wölfle S.J., Strebovsky J., Bartz H., Sähr A., Arnold C., Kaiser C., Dalpke A.H., Heeg K. (2011). PD-L1 expression on tolerogenic APCs is controlled by STAT-3. Eur. J. Immunol..

[30] Vasquez-Dunddel D., Pan F., Zeng Q., Gorbounov M., Albesiano E., Fu J., Blosser R.L., Tam A.J., Bruno T., Zhang H. (2013). STAT3 regulates arginase-I in myeloid-derived suppressor cells from cancer patients. J. Clin. Investig..

[31] Wu L., Du H., Li Y., Qu P., Yan C. (2011). Signal transducer and activator of transcription 3 (Stat3C) promotes myeloid-derived suppressor cell expansion and immune suppression during lung tumorigenesis. Am. J. Pathol..

[32] Melillo J.A., Song L., Bhagat G., Blazquez A.B., Plumlee C.R., Lee C., Berin C., Reizis B., Schindler C. (2010). Dendritic cell (DC)-specific targeting reveals Stat3 as a negative regulator of DC function. J. Immunol..

[33] Wang T., Niu G., Kortylewski M., Burdelya L., Shain K., Zhang S., Bhattacharya R., Gabrilovich D., Heller R., Coppola D. (2004). Regulation of the innate and adaptive immune responses by Stat-3 signaling in tumor cells. Nat. Med..

[34] Nefedova Y., Huang M., Kusmartsev S., Bhattacharya R., Cheng P., Salup R., Jove R., Gabrilovich D. (2004). Hyperactivation of STAT3 is involved in abnormal differentiation of dendritic cells in cancer. J. Immunol..

[35] Panopoulos A.D., Zhang L., Snow J.W., Jones D.M., Smith A.M., El Kasmi K.C., Liu F., Goldsmith M.A., Link D.C., Murray P.J. (2006). STAT3 governs distinct pathways in emergency granulopoiesis and mature neutrophils. Blood.

[36] Zhang H., Nguyen-Jackson H., Panopoulos A.D., Li H.S., Murray P.J., Watowich S.S. (2010). STAT3 controls myeloid progenitor growth during emergency granulopoiesis. Blood.

[37] Kortylewski M., Kujawski M., Wang T., Wei S., Zhang S., Pilon-Thomas S., Niu G., Kay H., Mulé J., Kerr W.G. (2005). Inhibiting Stat3 signaling in the hematopoietic system elicits multicomponent antitumor immunity. Nat. Med..

[38] Chen W., Jin W., Hardegen N., Lei K.J., Li L., Marinos N., McGrady G., Wahl S.M. (2003). Conversion of peripheral CD4+ CD25-naive T cells to CD4+ CD25+ regulatory T cells by TGF-beta induction of transcription factor Foxp3. J. Exp. Med..

[39] Hsu P., Santner-Nanan B., Hu M., Skarratt K., Lee C.H., Stormon M., Wong M., Fuller S.J., Nanan R. (2015). IL-10 Potentiates Differentiation of Human Induced Regulatory T Cells via STAT3 and Foxo1. J. Immunol..

[40] Kong L.Y., Wei J., Sharma A.K., Barr J., Abou-Ghazal M.K., Fokt I., Weinberg J., Rao G., Grimm E., Priebe W. (2009). A novel phosphorylated STAT3 inhibitor enhances T cell cytotoxicity against melanoma through inhibition of regulatory T cells. Cancer Immunol. Immunother..

[41] Chou W.-C., Levy D.E., Lee C.-K. (2006). STAT3 positively regulates an early step in B-cell development. Blood.

[42] Ding C., Chen X., Dascani P., Hu X., Bolli R., Zhang H.-G., McLeish K.R., Yan J. (2016). STAT3 Signaling in B Cells Is Critical for Germinal Center Maintenance and Contributes to the Pathogenesis of Murine Models of Lupus. J. Immunol..

[43] Yang C., Lee H., Pal S., Jove V., Deng J., Zhang W., Hoon D.S., Wakabayashi M., Forman S., Yu H. (2013). B cells promote tumor progression via STAT3 regulated-angiogenesis. PLoS ONE.

[44] Zhang C., Xin H., Zhang W., Yazaki P.J., Zhang Z., Le K., Li W., Lee H., Kwak L., Forman S. (2016). CD5 Binds to Interleukin-6 and Induces a Feed-Forward Loop with the Transcription Factor STAT3 in B Cells to Promote Cancer. Immunity.

[45] Zhou J., Min Z., Zhang D., Wang W., Marincola F., Wang X. (2014). Enhanced frequency and potential mechanism of B regulatory cells in patients with lung cancer. J. Transl. Med..

[46] Zhou X., Liu S., Liu J., Zhang Z., Mao X., Zhou H. (2020). MicroRNA-130a enhances the killing ability of natural killer cells against non-small cell lung cancer cells by targeting signal transducers and activators of transcription 3. Biochem. Biophys. Res. Commun..

[47] Yue C., Shen S., Deng J., Priceman S.J., Li W., Huang A., Yu H. (2015). STAT3 in CD8+ T Cells Inhibits Their Tumor Accumulation by Downregulating CXCR3/CXCL10 Axis. Cancer Immunol. Res..

[48] Oh H.M., Yu C.R., Golestaneh N., Amadi-Obi A., Lee Y.S., Eseonu A., Mahdi R.M., Egwuagu C.E. (2011). STAT3 protein promotes T-cell survival and inhibits interleukin-2 production through up-regulation of Class O Forkhead transcription factors. J. Biol. Chem..

[49] Schmetterer K.G., Neunkirchner A., Wojta-Stremayr D., Leitner J., Steinberger P., Pickl W.F. (2015). STAT3 governs hyporesponsiveness and granzyme B-dependent suppressive capacity in human CD4+ T cells. FASEB J..

[50] Yu X., Gao R., Li Y., Zeng C. (2020). Regulation of PD-1 in T cells for cancer immunotherapy. Eur. J. Pharmacol..

[51] Marin-Acevedo J.A., Kimbrough E.O., Lou Y. (2021). Next generation of immune checkpoint inhibitors and beyond. J. Hematol. Oncol..

[52] Pawelczyk K., Piotrowska A., Ciesielska U., Jablonska K., Gletzel-Plucinska N., Grzegrzolka J., Podhorska-Okolow M., Dziegiel P., Nowinska K. (2019). Role of PD-L1 Expression in Non-Small Cell Lung Cancer and Their Prognostic Significance according to Clinicopathological Factors and Diagnostic Markers. Int. J. Mol. Sci..

[53] Mino-Kenudson M. (2016). Programmed cell death ligand-1 (PD-L1) expression by immunohistochemistry: Could it be predictive and/or prognostic in non-small cell lung cancer?. Cancer Biol. Med..

[54] Mu C.Y., Huang J.A., Chen Y., Chen C., Zhang X.G. (2011). High expression of PD-L1 in lung cancer may contribute to poor prognosis and tumor cells immune escape through suppressing tumor infiltrating dendritic cells maturation. Med. Oncol..

[55] Mao Y., Li W., Chen K., Xie Y., Liu Q., Yao M., Duan W., Zhou X., Liang R., Tao M. (2015). B7-H1 and B7-H3 are independent predictors of poor prognosis in patients with non-small cell lung cancer. Oncotarget.

[56] Lee C.K., Man J., Lord S., Links M., Gebski V., Mok T., Yang J.C. (2017). Checkpoint Inhibitors in Metastatic EGFR-Mutated Non-Small Cell Lung Cancer-A Meta-Analysis. J. Thorac. Oncol..

[57] Gainor J.F., Shaw A.T., Sequist L.V., Fu X., Azzoli C.G., Piotrowska Z., Huynh T.G., Zhao L., Fulton L., Schultz K.R. (2016). EGFR Mutations and ALK Rearrangements Are Associated with Low Response Rates to PD-1 Pathway Blockade in Non-Small Cell Lung Cancer: A Retrospective Analysis. Clin. Cancer Res..

[58] Liu Z., Yu X., Xu L., Li Y., Zeng C. (2022). Current insight into the regulation of PD-L1 in cancer. Exp. Hematol. Oncol..

[59] Marzec M., Zhang Q., Goradia A., Raghunath P.N., Liu X., Paessler M., Wasik M.A. (2008). Oncogenic kinase NPM/ALK induces through STAT3 expression of immunosuppressive protein CD274 (PD-L1, B7-H1). Proc. Natl. Acad. Sci. USA.

[60] Hou J., Zhao R., Xia W., Chang C.W., You Y., Hsu J.M., Hung M.C. (2020). PD-L1-mediated gasdermin C expression switches apoptosis to pyroptosis in cancer cells and facilitates tumour necrosis. Nat. Cell Biol..

[61] Fang W., Zhang J., Hong S., Zhan J., Chen N., Qin T., Zhang L. (2014). EBV-driven LMP1 and IFN-gamma up-regulate PD-L1 in nasopharyngeal carcinoma: Implications for oncotargeted therapy. Oncotarget.

[62] He J., Zhang W., Di T., Meng J., Qi Y., Li G., Yan W. (2020). Water extract of sporoderm-broken spores of *Ganoderma lucidum* enhanced pd-l1 antibody efficiency through downregulation and relieved complications of pd-l1 monoclonal antibody. Biomed. Pharmacother..

[63] You W., Liu X., Yu Y., Chen C., Xiong Y., Liu Y., Li R. (2020). miR-502-5p affects gastric cancer progression by targeting PD-L1. Cancer Cell Int..

[64] Zhang N., Zeng Y., Du W., Zhu J., Shen D., Liu Z., Huang J.A. (2016). The EGFR pathway is involved in the regulation of PD-L1 expression via the IL-6/JAK/STAT3 signaling pathway in EGFR-mutated non-small cell lung cancer. Int. J. Oncol..

[65] Koh J., Jang J.-Y., Keam B., Kim S., Kim M.-Y., Go H., Kim T.M., Kim D.-W., Kim C.-W., Jeon Y.K. (2015). EML4-ALKenhances programmed cell death-ligand 1 expression in pulmonary adenocarcinoma via hypoxia-inducible factor (HIF)-1α and STAT3. Oncoimmunology.

[66] Roussel H., De Guillebon E., Biard L., Mandavit M., Gibault L., Fabre E., Antoine M., Hofman P., Beau-Faller M., Blons H. (2017). Composite biomarkers defined by multiparametric immunofluorescence analysis identify ALK-positive adeno-carcinoma as a potential target for immunotherapy. Oncoimmunology.

[67] Ota K., Azuma K., Kawahara A., Hattori S., Iwama E., Tanizaki J., Harada T., Matsumoto K., Takayama K., Takamori S. (2015). Induction of PD-L1 Expression by the EML4-ALK Oncoprotein and Downstream Signaling Pathways in Non-Small Cell Lung Cancer. Clin. Cancer Res..

[68] Sumimoto H., Takano A., Teramoto K., Daigo Y. (2016). RAS–Mitogen-Activated Protein Kinase Signal Is Required for Enhanced PD-L1 Expression in Human Lung Cancers. PLoS ONE.

[69] Tang D., Zhao D., Wu Y., Yao R., Zhou L., Lu L., Gao W., Sun Y. (2018). The miR-3127-5p/p-STAT3 axis up-regulates PD-L1 inducing chemoresistance in non-small-cell lung cancer. J. Cell. Mol. Med..

[70] Abdelhamed S., Ogura K., Yokoyama S., Saiki I., Hayakawa Y. (2016). AKT-STAT3 Pathway as a Downstream Target of EGFR Signaling to Regulate PD-L1 Expression on NSCLC cells. J. Cancer.

[71] Luo F., Luo M., Rong Q.X., Zhang H., Chen Z., Wang F., Zhao H.Y., Fu L.W. (2019). Niclosamide, an antihelmintic drug, enhances efficacy of PD-1/PD-L1 immune checkpoint blockade in non-small cell lung cancer. J. Immunother. Cancer.

[72] Pore N., Wu S., Standifer N., Jure-Kunkel M., de los Reyes M., Shrestha Y., Halpin R., Rothstein R., Mulgrew K., Blackmore S. (2021). Resistance to Durvalumab and Durvalumab plus Tremelimumab Is Associated with Functional STK11 Mutations in Patients with Non–Small Cell Lung Cancer and Is Reversed by STAT3 Knockdown. Cancer Discov..

[73] Herrmann A., Lahtz C., Nagao T., Song J.Y., Chan W.C., Lee H., Yue C., Look T., Mülfarth R., Li W. (2017). CTLA4 Promotes Tyk2-STAT3-Dependent B-cell Oncogenicity. Cancer Res..

[74] Austin J.W., Lu P., Majumder P., Ahmed R., Boss J.M. (2014). STAT3, STAT4, NFATc1, and CTCF regulate PD-1 through multiple novel regulatory regions in murine T cells. J. Immunol..

[75] Celada L.J., Kropski J.A., Herazo-Maya J.D., Luo W., Creecy A., Abad A.T., Chioma O.S., Lee G., Hassell N.E., Shaginurova G.I. (2018). PD-1 up-regulation on CD4(+) T cells promotes pulmonary fibrosis through STAT3-mediated IL-17A and TGF-β1 production. Sci. Transl. Med..

[76] Zaretsky J.M., Garcia-Diaz A., Shin D.S., Escuin-Ordinas H., Hugo W., Hu-Lieskovan S., Torrejon D.Y., Abril-Rodriguez G., Sandoval S., Barthly L. (2016). Mutations associated with acquired resistance to PD-1 blockade in melanoma. N. Engl. J. Med..

[77] Saigi M., Alburquerque-Bejar J.J., Mc Leer-Florin A., Pereira C., Pros E., Romero O.A., Baixeras N., Esteve-Codina A., Nadal E., Brambilla E. (2018). MET-oncogenic and JAK2-inactivating alterations are inde-pendent factors that affect regulation of PD-L1 expression in lung cancer. Clin. Cancer Res..

[78] Mitchell K.A., Nichols N., Tang W., Walling J., Stevenson H., Pineda M., Stefanescu R., Edelman D.C., Girvin A.T., Zingone A. (2019). Recurrent PTPRT/JAK2 mutations in lung adenocarcinoma among African Americans. Nat. Commun..

[79] Papavassiliou K.A., Zoi I., Gargalionis A.N., Koutsilieris M. (2019). Polycystin-1 affects cancer cell behaviour and interacts with mTOR and Jak signalling pathways in cancer cell lines. J. Cell. Mol. Med..

[80] Moutsatsou P., Papavassiliou A.G. (2008). The glucocorticoid receptor signalling in breast cancer. J. Cell. Mol. Med..

[81] Karamouzis M.V., Gorgoulis V.G., Papavassiliou A.G. (2002). Transcription factors and neoplasia: Vistas in novel drug design. Clin. Cancer Res..

[82] Papatsoris A.G., Karamouzis M.V., Papavassiliou A.G. (2005). Novel insights into the implication of the IGF-1 network in prostate cancer. Trends Mol. Med..

[83] Witt K., Evans-Axelsson S., Lundqvist A., Johansson M., Bjartell A., Hellsten R. (2021). Inhibition of STAT3 augments antitumor efficacy of anti-CTLA-4 treatment against prostate cancer. Cancer Immunol. Immunother..

[84] Shinozaki E., Kawazoe A., Kuboki Y., Komatsu Y., Nishina T., Hara H., Yuki S., Shitara K., Bando H., Kotani D. (2018). Multicenter phase I/II trial of BBI608 and pembrolizumab combination in patients with metastatic colorectal cancer (SCOOP study): EPOC1503. J. Clin. Oncol..

[85] Attili I., Karachaliou N., Bonanno L., Berenguer J., Bracht J., Codony-Servat J., Codony-Servat C., Ito M., Rosell R. (2018). STAT3 as a potential immunotherapy biomarker in oncogene-addicted non-small cell lung cancer. Ther. Adv. Med. Oncol..

[86] Karamouzis M.V., Likaki-Karatza E., Ravazoula P., Badra F.A., Koukouras D., Tzorakoleftherakis E., Papavassiliou A.G., Kalofonos H.P. (2002). Non-palpable breast carcinomas: Correlation of mammographically detected malignant-appearing microcalcifications and molecular prognostic factors. Int. J. Cancer.

[87] Shin S.U., Lee J., Kim J.H., Kim W.H., Song S.E., Chu A. (2017). Gene expression profiling of calcifications in breast cancer. Sci. Rep..

[88] Ji L., Jianli M., Qingyuan Z. (2022). Identification of the pyroptosis-related prognostic gene signature and characterization of tumor microenvironment infiltration in triple-negative breast cancer. Front. Genet..

[89] Konstantinopoulos P.A., Vandoros G.P., Papavassiliou A.G. (2006). FK228 (depsipeptide): A HDAC inhibitor with pleiotropic antitumor activities. Cancer Chemother. Pharmacol..

[90] Shi Y., Fu Y., Zhang X., Zhao G., Yao Y., Guo Y. (2021). Romidepsin (FK228) regulates the expression of the immune checkpoint ligand PD-L1 and suppresses cellular immune functions in colon cancer. Cancer Immunol. Immunother..

